# Micro-Current Stimulation Has Potential Effects of Hair Growth-Promotion on Human Hair Follicle-Derived Papilla Cells and Animal Model

**DOI:** 10.3390/ijms22094361

**Published:** 2021-04-22

**Authors:** Donghyun Hwang, Hana Lee, Jinho Lee, Minjoo Lee, Seungkwan Cho, Tackjoong Kim, Hansung Kim

**Affiliations:** 1 Department of Biomedical Engineering, Yonsei University, Wonju 26493, Korea; ggubuing@naver.com (D.H.); hanah4378@naver.com (H.L.); mj9732@naver.com (M.L.); 2 Division of Biological Science and Technology, Yonsei University, Wonju 26493, Korea; drlogos@naver.com (J.L.); ktj@yonsei.ac.kr (T.K.); 3 CELLOGIN Inc., Wonju 26354, Korea; biecsk@naver.com

**Keywords:** alopecia, hair growth, human hair follicle dermal papilla cell, micro-current stimulation

## Abstract

Recently, a variety of safe and effective non-pharmacological methods have been introduced as new treatments of alopecia. Micro-current electrical stimulation (MCS) is one of them. It is generally known to facilitate cell proliferation and differentiation and promote cell migration and ATP synthesis. This study aimed to investigate the hair growth-promoting effect of MCS on human hair follicle-derived papilla cells (HFDPC) and a telogenic mice model. We examined changes in cell proliferation, migration, and cell cycle progression with MCS-applied HFDPC. The changes of expression of the cell cycle regulatory proteins, molecules related to the PI3K/AKT/mTOR/Fox01 pathway and Wnt/β-catenin pathway were also examined by immunoblotting. Subsequently, we evaluated the various growth factors in developing hair follicles by RT-PCR in MCS-applied (MCS) mice model. From the results, the MCS-applied groups with specific levels showed effects on HFDPC proliferation and migration and promoted cell cycle progression and the expression of cell cycle-related proteins. Moreover, these levels significantly activated the Wnt/β-catenin pathway and PI3K/AKT/mTOR/Fox01 pathway. Various growth factors in developing hair follicles, including Wnts, FGFs, *IGF-1*, and *VEGF-B* except for *VEGF-A*, significantly increased in MCS-applied mice. Our results may confirm that MCS has hair growth-promoting effect on HFDPC as well as telogenic mice model, suggesting a potential treatment strategy for alopecia.

## 1. Introduction

Alopecia (hair loss) is a widespread disease for both male and female throughout the world. It is classified as a dermatological disorder, which can cause psychological stress, due to nutritional deficiencies, hormonal changes, and hair cycle disorder [1,2,3]. There are many substances, including minoxidil, finasteride, dutasteride, ketoconazole, spironolactone, and flutamide, that have been used for the treatment of alopecia, and only two drugs, minoxidil and finasteride, were approved by Food and Drug Administration (FDA) as hair growth drugs [4]. Due to its non-invasive properties and high absorption, the topical application using minoxidil and oral administration using finasteride are widely used for the prevention and treatment of hair loss. However, pharmacological treatments have been reported various side effects such as sexual dysfunction, hypertension, and fetal defects [5,6,7]. In this respect, it is necessary to develop the alternative methods with non-pharmacological approaches, which are effective on the prevention of hair loss and promotion of hair growth safely. Currently, several methods such as laser and electrical simulation have been suggested as the non-pharmacological treatments [8,9,10,11]. Among them, electrical stimulation is known to facilitate cell proliferation and differentiation, promote cell migration and ATP synthesis through the influx of calcium ions into cells, and activate protein synthesis mainly through PI3K- and Ca^2+^-related mechanisms. For this reason, it is believed that electrical stimulation can regulate the secretion of several hair growth factors to promote the proliferation of hair follicular dermal papilla cells, prolong the growth phase, and ultimately promote hair regeneration [12,13,14]. Although some studies revealed that electrical stimulation could be promising to induce hair growth, it is difficult to assess its clinical efficacy. This is because there is a lack of parameter study referring to cell behavior and mechanism of evaluation of electrical stimulation on hair growth.

Recent years have seen increased use of micro-current electrical stimulation (MCS) for successful therapy with a low total amount of current [15]. MCS is a low-current therapy with an intensity of 1 mA or less that utilizes the body’s physiological current (bio-current) and increases bioactivity to repair nerve, muscle, and tissue damage [16]. Cheng et al. reported that micro-current stimulation promotes cell repair through the formation of potential differences in cell membranes to open ion channels and transport Ca^2+^ ions into the cell, which increase adenosine triphosphate (ATP) production and protein synthesis by triggering chemical processes [12]. Furthermore, there is almost no discomfort or side effects since the amount of current used is within the range of bio-current [17]. This study was performed to evaluate the applicability of MCS as an alternative to pharmacotherapy for the treatment of alopecia.

The dermal papilla cells (DPCs) derived from dermal mesenchymal cells are specialized mesenchymal components of hair that play a very important role in controlling the hair growth cycle and hair follicle formation [18]. DPCs regulate hair follicle development by acting as a reservoir of multipotent stem cells, nutrients, and growth factors. Some researchers have suggested that functional abnormalities in the DPCs cause hair loss due to an imbalance in the hair follicle cycle. Other researchers also found that the number of DPCs during the anagen phase increases, producing signals that regulate keratinocyte proliferation and differentiation [19,20]. Because they play a crucial role in hair growth, DPCs have been widely used in numerous studies as in vitro screening model to evaluate the effect of hair-growth modulating agents at the cellular and molecular level [21].

In this study, we investigated which levels of MCS have pro-proliferative effect on human hair follicle-derived papilla cells (HFDPC) and whether selected levels of MCS consequently have induced the hair growth effect on animal model.

## 2. Results

### 2.1. MCS Stimulated HFDPC Cell Proliferation and Migration

**Figure 1:**
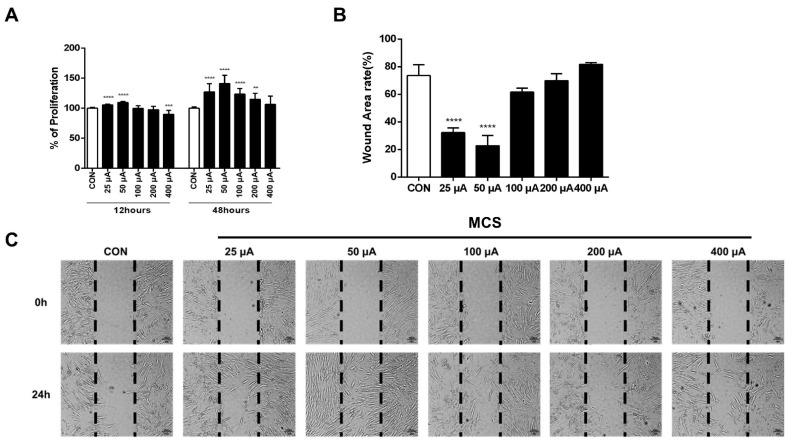
(**A**) Effects of MCS on the proliferation of HFDPC cells. To induce cell proliferation, MCS was applied to the HFDPC for 1 h, and the cell was subsequently cultured for 12 and 48 h and incubated further with WST-1 reagent for additional 1 h. The values shown represent the mean ± SD of triplicate measurements of separate experiments. Values are shown as percentages of the control. (**B**) Relative wound area rate was calculated as the ratio of the remaining wound area at 24 h and the original area at 0 h (**C**) In vitro scratch assay. Black dotted lines indicate the wound borders at the beginning of the assay and were recorded at 0 and 24 h post-scratching. HFDPC cells were treated with various levels of MCS or left untreated. ** *p* < 0.01, *** *p* < 0.001, **** *p* < 0.0001 vs. control group.

To investigate whether MCS could induce the proliferation of HFDPC, HFDPC cells were treated with various levels of MCS. The MCS of 25 μA, 50 μA, 100 μA, 200 μA, and 400 μA levels were applied for 1 h, and the colorimetric analysis was performed by WST-1 assay after 12 h and 24 h, respectively. The cell proliferation of the MCS groups 25 μA and 50 μA were significantly increased compared to that of the CON group. On the other hand, the level of cell proliferation in the 400 μA group was significantly decreased compared to that of the CON group at 12 h. However, cell proliferation in all MCS groups was higher than that of the CON group at 24 h (Figure 1A). To study the effects of MCS on cell migration, we performed scratch wound-healing assays. In this study, MCS was applied for 1 h on HFDPC cells just before incubating HFDPC cells, and migration was observed 24 h after the scratch. The wound area rate of 25 μA and 50 μA MCS groups were significantly lower than that of the control group. These results represented that the 25 μA and 50 μA groups showed more effects on HFDPC proliferation and migration compared with the control group or with the group treated with other MCS levels. For these reasons, 25 μA and 50 μA levels of currents were selected for analyzing the mechanism of MCS for hair growth (Figure 1B,C).

### 2.2. MCS Enhanced Cell Cycle Progression

**Figure 2:**
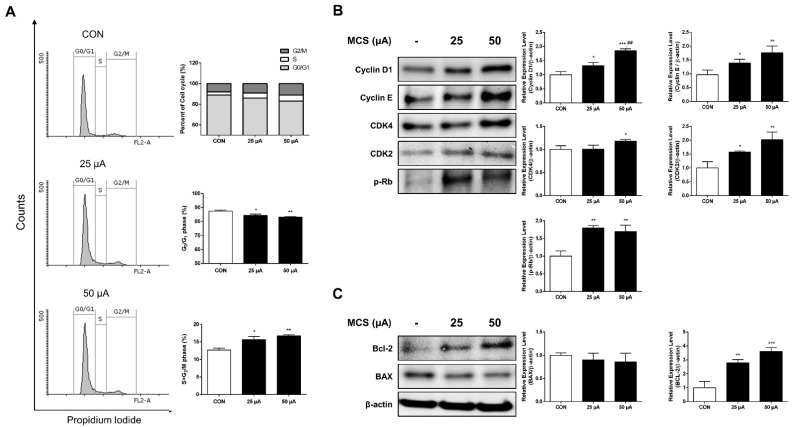
MCS groups promote cell cycle progression and inhibit cell apoptosis. (**A**) Change in cell cycle distribution in HFDPC following the treatment of MCS. HFDPC were treated with various levels of MCS for 24 h. The analysis of cell cycle distribution was performed by flow cytometry after the staining of DNA by propidium iodide. The population of cells in G0/G1, S, and G2/M phases in MCS-treated HFDPC. * *p* < 0.05, ** *p* < 0.001. (**B**) Immunoblot analysis of cell cycle-related proteins on HFDPC following the treatment of MCS. The cell lysate was analyzed by immunoblot using Cyclin D1, Cyclin E, CDK4, CDK2, and p-Rb antibodies. (**C**) Immunoblot analysis of cell apoptosis-related proteins on HFDPC following the treatment of MCS. The graph represents the quantitative level of the proteins. * *p* < 0.05, ** *p* < 0.01, *** *p* < 0.001 vs. control group; ## *p* < 0.01 vs. 25 μA MCS group.

To examine the effects of MCS on cell-cycle progression, HFDPC was investigated via flow cytometry for propidium iodide staining using FACS after the treatment of MCS. MCS groups decreased the percentage of cells in G0/G1 phase compared to the untreated control while the S phase and G2/M phase were increased by the treatment of MCS (Figure 2A). These data indicated that MCS promotes cell-cycle progression in HFDPC.

### 2.3. Effect of MCS on G1 Phase Related Protein and Apoptosis Related Protein Expression

Based on the results of cell cycle distribution, the expression of cell cycle regulatory proteins such as Cyclin D1, Cyclin E, CDK4, CDK2, and p-Rb, which are essential for cell cycle progression from G1 to S phase, were investigated. HFDPC was treated with 25 μA and 50 μA of MCS for 1 h to determine the level of these proteins by immunoblotting. Our results revealed that MCS groups significantly increased the protein levels of Cyclin D1, Cyclin E, CDK2, CDK4 (except 25 μA), and p-Rb (Figure 2B). The protein expression level of Bcl-2 was significantly increased compared to the CON group, and that of BAX was slightly decreased in the MCS groups (Figure 2C). These results suggest that MCS at the level of 25 μA and 50 μA induces HFDPC proliferation by enhancing the progression of the cell cycle phases and inhibiting cell apoptosis.

### 2.4. MCS-Induced Activation of the PI3K/AKT/mTOR/Fox01 Signaling Pathway and Wnt Pathway Affects Upregulation of β-Catenin Expression

**Figure 3:**
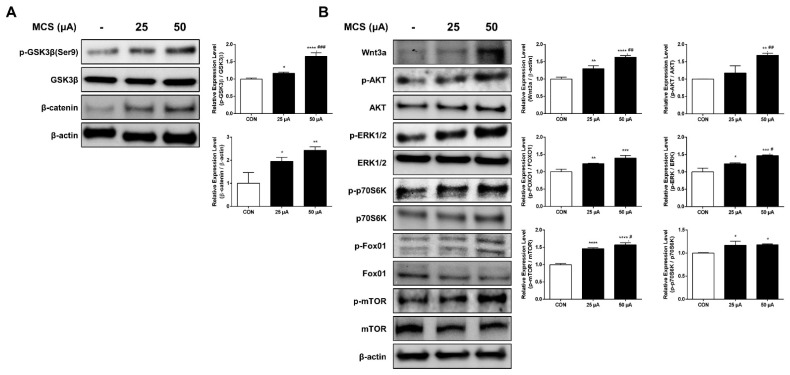
MCS upregulates the GSK3β/β-catenin signaling pathway and PI3K/AKT/mTOR/Fox01 signaling pathway. (**A**) Immunoblot analysis of the expression of p-GSK3β (Ser9), GSK3β, and β-catenin in HFDPC cells following treatment with MCS. (**B**) Immunoblot analysis of the expression of Wnt3a, p-AKT, AKT, p-ERK1/2, ERK1/2, p-p70S6K, p70S6K, p-Fox01, Fox01, p-mTOR, and mTOR in HFDPC cells following treatment with MCS. * *p* < 0.05, ** *p* < 0.01, *** *p* < 0.001, **** *p* < 0.0001 vs. control group; # *p* < 0.05, ## *p* < 0.01 vs. 25 μA MCS group.

To examine the changes in the expression levels of GSK3β and β-catenin, we evaluated the expression of proteins by immunoblotting. From the results, the total β-catenin expression and phosphorylation of GSK3β at ser9 in HFDPC were increased in MCS groups (Figure 3A). Moreover, the protein levels of p-AKT, p-ERK, p-mTOR, p-FOX01, and p-p70S6K were also increased after stimulation. In addition, the Wnt3a was significantly increased after MCS, which indicate that the Wnt/β-catenin pathway was activated (Figure 3B).

It is known that the β-catenin and GSK3β can modulate via the PI3K/AKT/mTOR/Fox01 signaling pathway and the Wnt pathway, which is involved in regulating the cell growth, proliferation, migration, and apoptosis [22,23,24,25,26]. Based on our results, we can estimate that MCS-induced activation of the PI3K/AKT/mTOR/Fox01 signaling pathway and the Wnt pathway affects the upregulation of β-catenin expression.

### 2.5. The Effect of MCS on Hair Growth in the Telogenic C57BL/6 Mice

**Figure 4:**
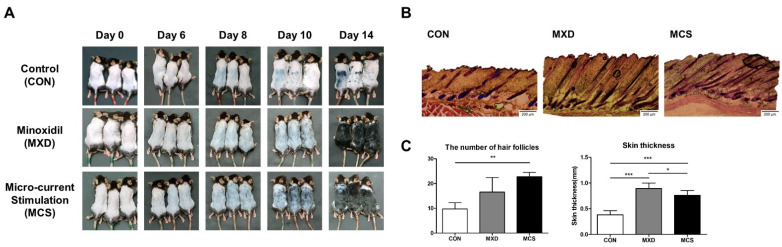
MCS accelerates the onset of black pigmentation and increases the number of follicles and skin thickness. (**A**) Photographs of shaved dorsal skin were taken at Day 0, 6, 8, 10 and 14 in control group (CON), minoxidil-treated group (MXD) used as a positive control, and micro-current stimulation with the intensity of 50 μA group (MCS). (**B**) Longitudinal sections of the dorsal skins for each group by H&E staining. (**C**) The number of hair follicles and skin thickness (Full-thickness) in the section for each group. * *p* < 0.05, ** *p* < 0.01, *** *p* < 0.001.

We evaluated the effect of MCS on visible hair growth for 14 days, and the dorsal skin of mice was recorded at day 0, 6, 8, 10, and 14 by the photo shown in Figure 4A. On day 6, black pigmentation started to show in the micro-current stimulation-applied group (MCS) and the minoxidil-treated group (MXD), and the MCS group showed more black pigmentation distribution than the MXD group. On day 8, the expression of black pigmentation started in the CON group, and it can be seen that visible hair growth started only in the MCS group. On day 10, hair growth started partially in the MXD group, but the MCS group showed the entire distribution of the hair growth. On day 14, at the end of the experiment, the MCS group and the MXD group showed similar states to unshaved hair, but the CON group showed that partial hair growth had just begun.

To investigate the hair follicles and skin thickness, H&E staining was performed at the end of the experiment shown in Figure 4B,C. Comparing the number of hair follicles per section area, the MXD group and the MCS group had 16.5 and 22.75, respectively, but only the MCS group showed a significant difference compared with the CON group, which had 9.75 (*p* < 0.05). Similar to the result of the number of follicles, both the MXD group and the MCS group were significantly thicker compared to the CON group (*p* < 0.01). In particular, the MXD group was thicker than the MCS group (*p* < 0.05).

### 2.6. The mRNA Expression Level of Hair Growth Promotion Associated Genes in Mouse Skin Tissue

**Figure 5:**
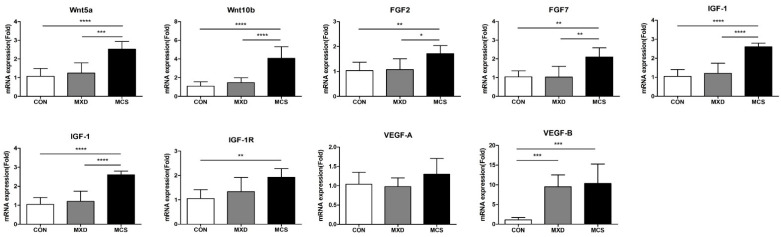
MCS increases the mRNA expression levels of growth factors contributing to telogen–anagen transition and hair growth promotion in telogenic mice. The mRNA expression levels were measured in the control group (CON), the minoxidil-treated group (MXD) used as a positive control, and micro-current stimulation with the intensity of the 50 μA group (MCS). All mRNA expression levels were normalized to that of *GAPDH* mRNA expression and expressed as fold changes relative to that of CON. * *p* < 0.05, ** *p* < 0.01, *** *p* < 0.001, **** *p* < 0.0001.

To determine whether MCS modulates the various growth factors in developing hair follicles and regulating the hair growth cycle, the expressions were measured at the mRNA levels in mouse skin tissue harvested after the experiment (Figure 5).

*Wnt5a* and *Wnt10b* mediate anagen conversion of the hair cycle expressed at a specific morphogenetic epidermal placode. Compared to the CON group, both mRNA levels of *Wnt5a* and *Wnt10b* showed no significant difference in the MXD group, but about 2.5 and 4.1 times of expression were shown in the MCS group (*p* < 0.0001). Similarly, FGFs are also known to induce the anagen phase in telogenic C57BL/6 mice, and these also tended to increase significantly only in the MCS group. *IGF-1* and *VEGF* are primarily present in the dermal papilla and modulate follicular growth via regulating proliferation of the follicular epithelium. Especially, *IGF-1* is fundamental to hair shaft differentiation in the development of hair follicles, and *IGF-1 receptor* (*IGF-1R*) may play a role in interacting with *IGF-1* for a proliferative and differentiative role in follicles. *IGF-1* gene expression was significantly increased only in the MCS group compared with the CON group (*p* < 0.0001); also, *IGF-1R* was increased in the MCS group (*p* < 0.01). In the MXD group used as the positive control group, there was no significant difference from the CON group. *VEGF* is namely known to be involved in hair growth by supplying nutrients to the hair follicle via angiogenesis. Its roles are known to affect the size and number of follicles and hair size. Concerning the *VEGF-A* gene expression, a major factor for angiogenesis, there was no significant difference among the groups. However, *VEGF-B*, which is critical for vascular survival, was increased both in the MXD group and in the MCS group compared with the CON group (*p* < 0.001).

## 3. Discussion

The human hair follicle dermal papilla cells (HFDPC) are responsible for the production of hair growth and development of hair follicles. The control of HFDPC proliferation is important in evaluating hair-growth treatments [22,23,24,27]. Currently, various studies have reported that low levels of electrical stimulation, particularly MCS, can play a role as an enhancer for cell proliferation [11,28]. This study was initiated to evaluate a hypothesis of whether MCS may enhance the HFDPC proliferation. However, as there have not been many previous studies on this, the known information is limited.

Meanwhile, unlike pharmacotherapy, physiotherapy, such as electrical stimulation, is often performed without a certain standard, and even when administered consistently, the response varies according to patient physical condition. Improper stimulation parameters can lead to results that are contrary to experimental expectations. For example, high intensity above 100 V/cm can cause cell membrane electroporation, an extreme increase in intracellular Ca^2+^ and reactive oxygen species, then induce cell apoptosis [29,30]. Besides, it has been consistently reported that there are limitations such as the heating effect in a high-frequency or a high current in the mA regime [31]. Therefore, to successfully treat alopecia utilizing electrical stimulation, it is important to first determine an appropriate current level.

The present study demonstrated that MCS of 50 µA particularly activated Wnt/β-catenin, ERK1/2, and the PI3K/AKT/mTOR/Fox01 signaling pathway and promoted cell cycle progression, which are involved in the proliferative and migration activity of HFDPC cells, compared to other MCS groups and the control group. From our results, the proliferation rate of HFDPC was significantly increased and the wound area rate was decreased in the 25 µA and 50 µA groups. For these reasons, 25 µA and 50 µA levels of currents were selected for analyzing the mechanism of MCS.

Cell proliferation is also determined by cell cycle transition regulation at specific points such as G1, S, and G2/M phases [25]. Cyclin D1, Cyclin E, CDK4, CDK2, and p-Rb are important proteins involved in cell proliferation and cell cycle. The Cyclin D1/CDK4 complexes are involved in the initiation of DNA synthesis, and near the end of the G1/S transition point, the expression of Cyclin E/CDK2 complexes that are required for the transition from G1 to S phase of the cell cycle that determines initiation of DNA duplication has been shown to promote the phosphorylation of Rb [26]. From the in-vitro results in this study, the portion of cells of the S phase and the G2/M phase of the cell cycle was increased by MCS. In the cell cycle analysis, the portion of cells of the S phase and the G2/M phase of the cell cycle was increased by MCS. The levels of Cyclin D1, CDK4, Cyclin E, CDK2, and p-Rb were increased by MCS.

Among the hair follicle cycle including anagen, catagen, telogen, and exogen, catagen is known as apoptosis-driven phase of regression [32]. In order to examine the inhibitory effect of MCS on HFDPC apoptosis, immunoblotting was performed. Bcl-2 and BAX proteins play an important role in apoptosis, and the decreased Bcl-2/BAX ratio leads the hair follicle to the anagen–catagen transition in hair cycle [33,34]. Bcl-2, an anti-apoptotic gene, plays an antagonistic role with BAX, a pro-apoptotic protein, in the apoptosis process [35]. The present study demonstrated that in the MCS group, the expression levels of Bcl-2 protein increased, and the expression levels of the BAX protein displayed no change. Taken together, these results suggest that MCS promotes cell proliferation by regulating cell cycle progression and inhibiting cell apoptosis.

In addition, we investigated whether MCS can modulate the PI3K/AKT/mTOR/Fox01 pathway and Wnt/β-catenin signaling known as the key signal pathway associated with hair growth. These contribute to various developmental and pathological processes, such as cell proliferation, cell differentiation, and cell migration in common [36,37,38,39,40,41]. If several growth factors activate the PI3K/AKT/mTOR/Fox01 pathway, this pathway controls various downstream AGC kinases, including AKT (phosphorylation on Ser473), serum- and glucocorticoid-stimulated kinase (SGK), and protein kinase Cα (PKCα). These modulate cell proliferation and cell growth via cytoskeleton regulation [42]. In particular, cell migration is known to be stimulated by the activation of PI3K/AKT/mTOR/Fox01 pathway and also play a critical role for hair follicle growth [43,44,45]. As seen Figure 1, cell migration was enhanced by 25 μA and 50 μA levels of MCS, and we found that MCS increased phosphorylation of AKT, mTOR, p70S6K, Fox01, and ERK1/2 [46]. In addition, Wnts play an important role in various aspects of hair follicle development, such as regeneration and maintenance of the anagen phase, and is considered to be an important signaling pathway for proliferation and differentiation in hair follicle cells [22,47,48,49]. When Wnts bind to the frizzled receptor and LRP5/6 co-receptors and activates the Wnt/β-catenin pathway, axin is separated from the complex and the activity of GSK3β kinase is inhibited, thereby stabilizing β-catenin. According to the recent study, it is reported that the treatment with a high level of Wnt3a and -7b significantly increased the cell proliferation by activating the expression of β-catenin and the activation of transcription factors including Axin2 and Lef1 [50].

Various studies have shown that β-catenin plays an essential role in maintaining hair growth. β-catenin is strongly expressed during the anagen phase in the DP and in the outer root sheath and the absence of β-catenin leads to the catagen phase [36,51,52,53]. In addition, β-catenin is considered to be an important signaling pathway for proliferation and differentiation in hair follicle cells [22,47,48,49]. Lacking β-catenin in keratinocyte downregulated hair cycle progression and differentiation of keratinocyte in hair follicles. The upregulation of stabilized β-catenin in prominin-1/CD133-positive DPC increases the proliferation of DPC as well as increases [49]. β-catenin is mainly controlled by the phosphorylation of casein kinase I and GSK3β in a complex consisting of GSK3β, Casein kinase I, and axin, and is degraded through the ubiquitination/proteasome pathway [54]. In mammalian cells, the activation of PI3K/AKT/mTOR/Fox01 signaling pathway upregulates β-catenin expression and initiate cell cycle progression by the inactivation of GSK3β, also known as a mediator of Wnt/β-catenin signaling [36,46,55,56]. Although it is implied that PI3K/AKT/mTOR/Fox01 signaling and Wnt/β-catenin signaling has commonality in that these modulate the interaction of β-catenin and GSK3β, these are not necessarily fully correlated until further research is undertaken as to whether GSK3β or β-catenin is a molecular cross-talk.

Our results showed that treatment with MCS leads to the phosphorylation of GSK3β at ser-9, which means inactivating GSK3β, and then induces β-catenin stabilization and accumulation (Figure 3A). It may be related to the activation of the PI3K/AKT/mTOR/Fox01 pathway, and the Wnt/β-catenin signaling pathway induced MCS as shown in Figure 3B. Moreover, it is reported that the degradation of Cyclin D1 is inhibited by Wnt/β-catenin signaling [55]. Correspondingly, our study showed that MCS activates the expression of Cyclin D1 as seen Figure 2B. Taken together, these results suggest that activated PI3K/Akt/mTOR/Fox01 pathway and Wnt/β-catenin signaling pathway by MCS may enhance the proliferation and migration of HFDPC, which are involved in hair growth. [57,58].

In order to analyze the effects of micro-current stimulation on hair growth-promoting activity with a telogenic C57BL/6 mice model, all animals were shaved and randomly assigned to three groups: Control (CON), Minoxidil-treated group (MXD) used as a positive control, and micro-current stimulation group (MCS). Comparing the onset of black pigmentation through the appearance of dorsal skin for each group, there is earlier onset in the MCS and the MXD groups on day 6 compared to the CON group on day 10. Black pigmentation represents an indicator of the transition of hair follicles from the telogen to the anagen phase, which might be compared with the distribution of black coloration [59], and both the MCS and the MXD groups showed promoting effects on this transition compared to the CON group. However, the visual hair shaft appeared on day 8 in the MCS group and began on day 10 in the MXD group, suggesting that hair growth promotion in anagen was more effective in the MCS group. In the H&E staining results on Day 14, there is a significant increase in the number of follicles in the MCS group only, but the skin thickness showed that both the MCS and the MXD groups were thickened without any edema or inflammation. The increased skin thickness is known to contribute indirectly the telogen to anagen transition, indicating a tendency similar to the number of hair follicles [50,60,61,62]. However, in the MXD group, hair follicle formation tended to be slower than in the MCS group, which probably differed in the growth factor contributing to hair follicle formation.

MCS might be expected to effective in promoting hair growth depending on the aforementioned results of black pigmentation, visible hair growth, the number of hair follicles, and skin thickness. To determine how MCS affects hair-growth-promotion-associated genes, such as the telogen-to-anagen transition or hair follicle number, we analyzed the mRNA expression level in dorsal skin for various growth factors.

Regarding the hair growth promotion, many crucial Wnt molecules including *Wnt5a*, *10a*, and *10b* are generally expressed at early morphogenetic stages [63]. Especially, *Wnt10b* is a major activator of the telogen–anagen transition, and it promoted hair growth via the Wnt/β-catenin signaling pathway [64,65]. Taken together, activation of Wnts on dorsal skin and activation of the Wnt/β-catenin signaling pathway by MCS-applied cell, demonstrate that MCS might accelerate the onset of black pigmentation in animals.

FGFs (*FGF2*, *FGF7*, and *FGF10*) have been reported to increase the number and size of hair follicles via promoting cell cycle and proliferation [59]. In the MCS group, all FGFs expression levels are upregulated compared to the CON and the MXD groups, which correspond to the previous result about the number of follicles. Moreover, *FGF10* appears with the highest efficiency among FGFs and causes increasing nuclear localization of β-catenin [39,59,66,67]. It might be a reason for the upregulation of β-catenin expression and cell proliferation in the MCS-applied cell previously shown.

While it is important to accelerate the telogen–anagen transition and increase the number of hair follicles, the prolongation of the anagen phase is also important for hair growth promotion. *IGF-1* is an essential growth factor for this prolongation as well as hair shaft differentiation [68]. Recent evidence suggests that these effects may be achieved by the interaction between *IGF-1* and *IGF-1R*, and it acts on anagen extension and catagen inhibition [69,70,71]. As a result of comparing the mRNA levels of *IGF-1* and *IGF-1R*, only the MCS group showed a significant increase in both genes, and these results would have caused an earlier appearance of the hair shaft in the MCS group.

However, among the various growth factor we analyzed, there is no difference in only *VEGF-A* expression from all groups. *VEGF-A* is known to play a key role in hair growth by supplying nutrients to the hair follicle and increasing follicle size [61,72]. Although this study has not shown the data regarding follicle size in each group, it can be seen that each group had a similar follicle size as shown in Figure 4B. On the other hand, *VEGF-B* expressions were increased by 9.5-fold expression in the MXD group and 10.3-fold expression in the MCS group compared to the Con group. Many studies found that *VEGF-B* is not an angiogenic factor but is critical for vascular survival factor [73,74,75,76]. Although it is not known to be directly related to hair growth promotion, it would be worth further study that why the *VEGF-B* expression level increased so much in both the MXD and the MCS groups.

## 4. Materials and Methods

### 4.1. Materials

HFDPCs were purchased from Promocell (Sickingenstr, Heidelberg, Germany). Dulbecco’s modified Eagle’s medium (DMEM), penicillin-streptomycin (PS), Fetal bovine serum (FBS), and Propidium iodide were purchased from Sigma-Aldrich Chemical Co. (St. Louis, MO, USA). EZ-Cytox cell viability assay kit was purchased from the Daeil Labservice (Seoul, Korea). PRO-PREP™ Protein Extraction Solution was purchased from iNtRON Biotechnology Inc. (Gyeonggi, Korea). Protease and Phosphatase Inhibitor Mini Tablets and BCA assay kit were purchased from Thermo Scientific (Rockford, IL, USA).

### 4.2. Design and Implementation of the Custom-Made Microcurrent Stimulating Chanmber System

**Figure 6:**
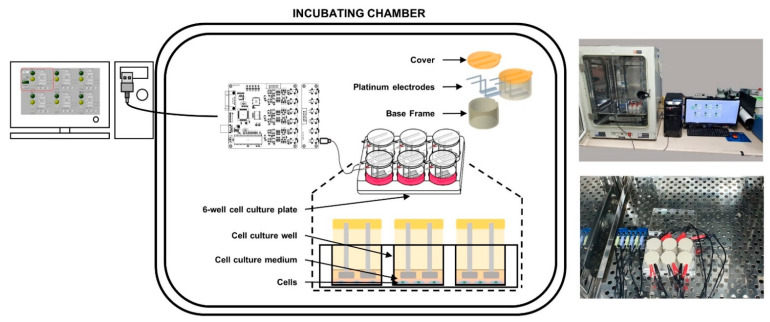
MCS system available in incubating chamber.

The stimulating system was fabricated to incubate HFDPCs in chamber simultaneously during application of MCS (Figure 6) (Yonsei University & CELLOGIN Inc., Gangwon, Korea). This system can be adjustable to apply current-based electric stimulation from 0 to 1000 μA, which has 5 μA of the unit interval. The type of pulse can adjust to the two modes, which include mono and biphasic pulsatile square pulses. The adjustable frequencies are from 0 to 500 Hz with 1 Hz of the unit interval. In this system, the condition of stimulating currents can be generated alternately in each separated culture wells.

The system was composed of a current controller, 6 sets of body frames, and a 6-well cell culture plate (diameter 35 mm). The set of body frames is comprised of a base frame, a cover, and 2 platinum electrodes per well. The electrode tip was a rectangular shape and was set to the size (7.5 mm × 22 mm × 1 mm) that MCS can reach and maintain throughout the entire well.

### 4.3. Application of MCS for In-Vitro and In-Vivo Experiment

In the in vitro experiment, the applied parameters are set to compartmental intensities with biphasic pulsatile square pulse and a frequency of 10 Hz to compare the effects of different intensities of MCS. The applied intensities were 25 μA, 50 μA, 100 μA, 200 μA, and 400 μA per whole culture well for 60 min. Meanwhile, the generated pulse was designed to maintain a specific pre-set intensity regardless of the change in the resistance through the whole closed circuit (electrode tip- cell culture medium-electrode tip). This is feasible to detect the skin impedance and adjust the input accordingly to induce the pre-set value [77]. Therefore, the applied intensities were not indicated by surface unit of electrode but defined as the values of intensity itself.

Moreover, according to the study by Yoshiyuki et al. [78], since electrical stimulation delivered at a frequency of more than 10 Hz may suppress the proliferation of human dermal fibroblasts, the frequency was fixed 10 Hz as a minimized effective range.

Subsequently, an in vivo animal experiment was conducted using 50 μA whole closed-loop (electrode tip-skin-electrode tip) that was relatively effective on the cell proliferation from the in-vitro experiment. To apply electrical stimulation to the animal, the custom-made electrodes made of conductive metal and hydrogels were attached to the back of the animals, applying 20 min of MCS each day for 14 days. The pulsed type and frequency were set to the same conditions as in-vitro experiment.

### 4.4. HFDPC Cell Culture

HFDPC were cultured at 37 °C in a humidified incubator containing 5% CO_2_ using DMEM containing 10% FBS and 1% PS as a growth medium.

### 4.5. Cell Viability

After the MCS of 25 μA, 50 μA, 100 μA, 200 μA, and 400 μA levels were applied to 6-well plates, the colorimetric analysis was performed by WST-1 assay after 12 h and 24 h. Cells were incubated for 1 h, and the growth medium without the addition of the WST solution was set to blank. Cell viability was measured at 450 nm using a microplate reader (Epoch, BioTek Instruments, Winooski, VT, USA).

### 4.6. Cell Migration Assay

Cell migration was accessed using a wound-healing assay to check the migration of cells. After the wells were fully filled with the cells, the cells were wounded with a scratcher, which can generate cell-free gap of 500 μm thick wall (SPL, Pyeongtaek, Korea) and washed with 1 × PBS to remove cell debris. After that, the cells were treated with MCS for 1 h after 24 h of starvation with cultured in a serum-free medium to suppress cell proliferation. After 24 h of incubation under serum-free conditions, the wound fields were observed to quantify wound area rate using a light microscope (ECLIPSE, TS2, Nikon, Tokyo, Japan).

### 4.7. Flow Cytometry Analysis

HFDPCs were harvested, fixed in 100% ethanol at 4 °C, then were washed twice with PBS and incubated with 10 μg/mL RNase A (Invitrogen, CA, USA) and 50 μg/mL propidium iodide solution for 30 min at 37 °C. Fluorescence from 1.0 × 10^4^ cells was captured by flow cytometry (BD Bioscience, San Jose, CA, USA). The percentage of cells in the G0/G1, S, and G2/M phases of the cell cycle peak was calculated using the Flowing Software v2 (University of Turku, Turku, Finland).

### 4.8. Immunoblotting

Equal amounts of protein were separated by SDS-PAGE gels and then transferred onto the PVDF membrane, blocked using 3% Blotting Grade Blocker in Tris-buffered Saline-Tween 20 (TBS-T) for 1 h, and then incubated overnight with the specific antibodies at 4 °C. In this experiment, primary antibodies were used including: Bcl-2 (#3498), Bax (#2772), Cyclin E1 (#4129), CDK2 (#2546), CDK4 (#2906), p-Rb (#9308), p-AKT (#9271), AKT (#4691), p-ERK1/2 (#4377), ERK1/2 (#4695), p-Fox01 (#9461), Fox01 (#2880), p70S6K (#9234), p-p70S6K (#2708), Wnt3a (#2721), β-Catenin (#9562), p-GSK3β (#5558) GSK3β (#12456), and β-actin (#4967) purchased from Cell Signaling Technology and Cyclin D1 (ab134175) purchased from abcam. The membranes were washed and incubated with anti-Rabbit IgG, HRP-linked Secondary Antibody (#7074, Cell Signaling Technology) for 1 h at RT, and specific protein bands were presented with Amersham™ ECL™ Prime Western Blotting Detection Reagent (RPN2236, GE Healthcare) and determined using Image Quant LAS 500 (GE healthcare, UK). Band densities were quantified using Image J software (1.52a version, National Institutes of Health, Bethesda, MD, USA).

### 4.9. Animals

Twenty-four 6-week-old male C57BL/6J mice (22.67 ± 1.7 g) were randomly assigned to three groups: Control group (CON), Minoxidil treated group (MXD), 50 μA MCS treated group (MCS). After, the depilatory Niclean (Ildong Pharmaceutical Co., Ltd., Seoul, Korea) was applied on the back of mice for shaving. Animals were given 200 μL of each sample (Control, Minoxidil), applied to the depilated area of each mouse once a day for 14 days. (Control; PBS, Minoxidil; 5% minoxidil solution, MCS; PBS + 50 μA MCS)

All animals were housed at 23 ± 3 °C and 50 ± 10% humidity with normal chow and water ad libitum. The experiments were approved by the Yonsei University Animal Care Committee (YWCI-202007-013-01) and conducted in accordance with the Guidelines for Animal Experimentation the National Institutes of Health guide for the care and use of Laboratory animals.

### 4.10. Histologic Evaluation

Dorsal skin lesions were obtained from mice in all groups (Control, MXD and MCS) and were fixed in 10% formalin and dehydrated in 10%, 15%, and 20% sucrose solution. In order to embed fixed skins in a cryomold, optimal cutting temperature (OCT) compound (FSC 22 Clear, Leica Biosystems, Wetzlar, Germany) was used. The tissues were sectioned and stained with hematoxylin and eosinTo measure the thickness of the tissues, tissues were visualized using microscope (Olympus DP80, Olympus Optical Co., Ltd., Tokyo, Japan) and were analyzed with image software (Cell Sens 1.8, Olympus Corporation, Munster, Germany). The skin thickness was defined as the distance from the epidermal granular layer to the upper edge of the panniculus carnosus. Measurements were performed in three fields of a layer, and their average values per mouse were expressed in millimeters.

### 4.11. Real-Time Reverse Transcription PCR (qRT-PCR)

Total RNA was isolated from tissues using TRIzol™ reagent (Invitrogen; Thermo Fisher Scientific, Inc., Waltham, MA, USA) according to the manufacturer’s instructions. The concentration of total RNA was determined by a Colibri Microvolume spectrophotometer (Titertek Berthold, Pforzheim, Germany). Reverse transcription of total RNA was performed using a cDNA Synthesis Kit (Takara Bio, Shiga, Japan). Quantitative real-time PCR analysis was performed using SYBR Green I and a Lightcycler ^®^ 96 instrument (Roche, Basel, Switzerland). The PCR conditions were 95 °C for 10 min, followed by 45 cycles of amplification (95 °C for 10 s, 55 °C for 10 s, and 72 °C for 10 s). The Cq value for each reaction was determined by the software (the LightCycler 96 SW1.1, Roche, Basel, Switzerland). The expression levels of genes were normalized to *GAPDH*. The primers used are presented in Table 1.

### 4.12. Statistical Analysis

All results were presented as the means ± SD. The statistical software package SPSS 25 (IBM SPSS Statistics, SPSS Inc., Chicago, IL, USA) was used to evaluate the effects of MCS. The statistical analysis was determined by one-way analysis of variance (ANOVA) followed by Tukey’s test. Differences were considered to be significant for values of *p* < 0.05.

## 5. Conclusions

Our results showed that MCS with the intensity of 50 μA can particularly promote cell proliferation and migration through modulating cell cycle progression as well as cell apoptosis. We also find that MCS activates PI3K/AKT/mTOR/Fox01 and Wnt/β-catenin signaling, which are reported to contribute to promoting cell proliferation and migration. Additionally, various growth factors, which contribute to the telogen–anagen transition and hair growth promotion in anagen, increased in the MCS-applied telogenic mice model. Taken together, these results suggest that MCS with the intensity of 50 μA might be a useful method for hair loss prevention and hair growth promotion.

## Figures and Tables

**Table 1:** Primer sequence and PCR conditions.

Species	Primer Name	Forward	Reverse
*Mouse*	*IGF-1*	GTCGTCTTCACACCTCTTCTACCT	GCACAGTACATCTCCAGTCTCCT
	*IGF-1R*	CTCAGGCTTCATCCGCAACAG	GTTCTCCAACTCCGAGGCAATG
	*Wnt5a*	CTGGCAGGACTTTCTCAAGG	CTCTAGCGTCCACGAACTCC
	*Wnt10b*	CCTGTCCGGACTGAGTAAGC	TTGCTCACCACTACCCTTCC
	*FGF2*	CAAGAACGGCGGCTTCTTC	GAAAGAAACAGTATGGCCT
	*FGF7*	AGACTGTTCTGTCGCACC	CCGCTGTGTGTCCATTTAG
	*FGF10*	TGTCCGCTGGAGAAGGCTGTTC	CTATGTTTGGATCGTCATGG
	*VEGF-A*	CGAGATAGAGTACATCTTCAAGCC	TCATCGTTACAGCAGCCTGC
	*VEGF-B*	AAAAAAAAAGGAGAGTGCTGTGAAG	TCCCAGCCCGGAACAGA
	*GAPDH*	GCCAAGGTCATCCATGACAACT	GAGGGGCCATCCACAGTCTT

## Data Availability

Not applicable.
